# Screening and Evaluation of Excellent Blackberry Cultivars and Strains Based on Nutritional Quality, Antioxidant Properties, and Genetic Diversity

**DOI:** 10.3390/plants12162982

**Published:** 2023-08-18

**Authors:** Huifang Zhao, Yaqiong Wu, Wenlong Wu, Weilin Li, Yongcan Jin

**Affiliations:** 1 Co-Innovation Center for Sustainable Forestry in Southern China, College of Forestry, Nanjing Forestry University, 159 Longpan Road, Nanjing 210037, China; zhaohuifang@jib.ac.cn; 2 Institute of Botany, Jiangsu Province and Chinese Academy of Sciences (Nanjing Botanical Garden Memorial Sun Yat-Sen), Jiangsu Key Laboratory for the Research and Utilization of Plant Resources, Nanjing 210014, China; yqwu@cnbg.net (Y.W.); wuwenlong@jib.ac.cn (W.W.); 3 Jiangsu Co-Innovation Center of Efficient Processing and Utilization of Forest Resources, College of Light Industry and Food Engineering, Nanjing Forestry University, Nanjing 210037, China

**Keywords:** growth, fruit quality, cluster analysis, principal component analysis, SSR molecular markers, fingerprint

## Abstract

To screen and evaluate excellent blackberry cultivars and strains, 17 indexes of plant growth and fruit horticultural and nutritional characteristics were measured, 20 simple sequence repeat (SSR) markers were analyzed, the fingerprints of 23 blackberry cultivars and strains were constructed, and the processing characteristics of 10 excellent cultivars and strains were evaluated. The results showed that ‘Chester’ and ‘Shuofeng’ had the highest plant yield (6.5 kg per plant), of which the ‘Chester’ fruit also had the highest hardness (2.78 kg/cm^2^). ‘Kiowa’ had the highest single fruit weight (10.43 g). ‘10-5n-2’ had the highest total anthocyanin content (225.4 mg/100 g FW) and total polyphenol content (3.24 mg/g FW), but a low plant yield. These results suggest that ‘Shuofeng’ and ‘Chester’ are the top two blackberry cultivars planted in Nanjing, with the best growth and comprehensive quality. Moreover, a total of 119 alleles were detected with an average number of 6 alleles per locus. The polymorphism information content (PIC) was 0.374~0.844, with an average of 0.739, indicating a high genetic diversity among the 23 blackberry cultivars and strains. This study provides insight into the plant growth, fruit characteristics and genetic diversity of the 23 blackberry cultivars and strains, and is thus conducive to the protection and utilization of blackberry cultivars and strains.

## 1. Introduction

Blackberry (*Rubus* spp.) is native to North America and Europe and was introduced to China by the Institute of Botany, Jiangsu Province, and the Chinese Academy of Sciences (Nanjing Botanical Garden Mem. Sun Yat-Sen) in 1986. After more than 30 years of cultivation and promotion, it has become a characteristic agricultural industry in China, which has made great contributions to the development of low mountain and hilly areas and increased farmers’ incomes [1]. At present, blackberry has also become an important fruit tree suitable for growing in the low mountain and hilly areas of the middle and lower reaches of the Yangtze River in China [2,3]. Blackberry fruit is soft and juicy, with a bright color, refreshing sweet and sour taste, and unique flavor. It contains rich nutrients such as sugar, organic acids, and protein. In addition, blackberry fruits are rich in anthocyanins, flavonoids, polyphenols, and other secondary metabolites as beneficial bioactive ingredients [4], which have strong antioxidant, antiaging, and anticancer properties [5], and they can help prevent arteriosclerosis [6] and reverse diabetes and its complications [7].

However, in recent years, the main blackberry cultivars used in China have generally been the same, with the fruit maturity period concentrated in midsummer. This coincides with the high temperature and rainy season in the middle and lower reaches of the Yangtze River, and the berries are prone to decay and deterioration. Therefore, it is important to evaluate and screen the growth characteristics and fruit quality of existing blackberry cultivars and to breed new cultivars. First, the growth and maturity characteristics of cultivars or strains should be investigated [8]. Second, as an economic fruit tree, the screening of blackberry fruit appearance, quality, and processing characteristics is also a very important step [9]. On this basis, molecular biology techniques are combined to analyze the genetic diversity among cultivars and strains, construct their fingerprint maps, further clarify the genetic relationships between cultivars and strains [10], and comprehensively evaluate blackberry cultivars and strains based on phenotype, physiology, and genotype.

In terms of cultivating new cultivars, the research team has also carried out hybrid breeding work in recent years [11,12,13]. Through a series of steps such as raising seedlings, cultivation, and planting trials, a batch of high-quality blackberry cultivars has been obtained, including ‘Ningzhi 1’ [14], ‘Ningzhi 2’ [15], ‘Ningzhi 3’ [16,17], ‘Shuofeng’ [18], ‘Zaohong’ [19], ‘Shuofeng 2’, and ‘Wanfeng’, and the promotion and planting of these cultivars have been gradually carried out.

Polyphenols and anthocyanins are the two most important active antioxidant ingredients in blackberry fruit in addition to sugar and acid [20,21]. In recent years, with consumers’ increasing attention toward healthy foods, a comprehensive examination of the sugar, acid, anthocyanin, and polyphenol components of fruits is often conducted to evaluate fruit quality. Blackberry is a typically processed fruit due to its soft and juicy nature, lack of resistance to transportation and storage, and high acidity. Therefore, based on the fruit’s appearance, nutritional characteristics, and genetic characteristics, this study also investigated and evaluated the processing characteristics of blackberry fruit.

Second generation PCR-based molecular marker systems represented by simple sequence repeats (SSRs) and third generation DNA-based molecular marker systems represented by single nucleotide polymorphism (SNP) have been used in the identification of *Rubus* spp. [22,23], genetic diversity analysis [24], and fruit quality regulation [25,26]. Due to their good stability, strong reliability, and rich polymorphisms, simple sequence repeats (SSRs) are widely used in the analysis and evaluation of biological population genetic diversity [27,28]. Moreno-Medina et al. [23] used 16 pairs of SSR markers to evaluate the genetic diversity of 3 wild and 10 cultivated varieties of *Rubus* in Colombia and amplified 23 loci and 26 alleles. The polymorphism differences found in 76% of the samples arose from differences between the cultivated and wild species. Abdi et al. [27] used ISSR markers to study the genetic diversity of 45 blackberry genotypes (thorny and thornless) from the Surrey University of Agricultural Sciences and Natural Resources (SANRU) in the southern Caspian Sea and found that 10 pairs of ISSR primers amplified 345 fragments, of which 344 were polymorphic.

In this study, 23 blackberry cultivars and strains were selected as the research materials. The 23 blackberry cultivars and strains included 14 introduced cultivars, 7 newly bred cultivars, and 2 hybrid strains. Both the newly bred cultivars and strains had no thorns, while 7 of the 14 introduced cultivars were thorny (Table 1). To provide a reference for the introduction and promotion, quality identification, and resource protection of blackberries, the three research objectives of this article were as follows: to (1) comprehensively evaluate the growth and fruit quality of 23 blackberry cultivars and strains in the Nanjing region of China; (2) analyze the genetic diversity and construct DNA fingerprints of the 23 blackberry cultivars and strains using SSR markers; and (3) explore the processing characteristics of the fruits of 10 representative blackberry cultivars and strains.

## 2. Results

### 2.1. Analysis of the Growth Statistics of Different Blackberry Cultivars and Strains

Through comparative analysis, it was found that the number of basal branches of the 23 blackberry cultivars and strains ranged from 1 to 4, with an average of 2.4 (Table 1). The cultivars with the largest number of basal branches were ‘Arapaho’ (3.6) and ‘Boysen’ (3.2), and the cultivar with the least was ‘Shuofeng’ (1.4). The diameter of the basal branches of the 23 blackberry cultivars and strains ranged from 1.20~2.41 cm, with an average of 1.81 cm. The largest basal branch diameters were approximately 2.41 cm in ‘Chester’ and 2.40 cm in ‘Kiowa’, and the smallest was approximately 1.20 cm in ‘Black butte’. The number of effective branches of the 23 cultivars and strains was counted, and the number of effective branches of ‘Chester’ was the highest with up to 30, while ‘Kiowa’ had the next highest. ‘Black butte’ and ‘Young’ had the lowest number of effective branches, with approximately 10. The coefficient of variation of effective branching was the highest among the five growth indexes, reaching 25.6%. The diameter of the effective branches of ‘Triple Crown’ was the largest, up to 0.88 cm, and that of ‘Black Butte’ was the smallest at only 0.60 cm. In addition, the length of effective branches was different among the 23 cultivars and strains. ‘Shuofeng’ had the longest effective branches, up to 135 cm, and ‘Arapaho’ had the shortest effective branches, only 56 cm.

### 2.2. Comparison of Fruit Ripening Characteristics and Yield of Different Blackberry Cultivars and Strains

The investigation of the ripening characteristics of the different blackberry cultivars and strains showed that 11 of the 23 cultivars and strains were early-maturing cultivars (including 6 thornless cultivars), 6 were medium-maturing cultivars (including 5 thornless cultivars), and 6 were late-maturing cultivars and strains (including 2 thornless cultivars and 2 thornless hybrid strains). The maturity duration also varied significantly among cultivars and strains, with the shortest being approximately 20 days such as ‘Black Butte’, ‘Young’, and ‘Zaohei’, and the longest being 45 days such as ‘Kiowa’. The maturity duration of ‘Chester’ and ‘Wanfeng’ was 40 and 35 days, respectively, next to ‘Kiowa’, while that of the remaining 12 cultivars and strains were 30 days. The intervarietal variation coefficient of maturity duration was 20.2%.

Plant yield is influenced by growth statistics and maturity duration. Among the 23 cultivars and strains, ‘Black Butte’ had the smallest plant yield (1.3 kg), ‘Chester’ and ‘Shuofeng’ had the highest plant yield of approximately 6.5 kg, and ‘Kiowa’ and ‘Shuofeng 2’ had a high plant yield of more than 6.0 kg. The coefficient of variation of plant yield was the highest, up to 34.4%, among all the growth and maturity indexes (Table 1).

### 2.3. Comparison of Fruit Appearance Characteristics of Different Blackberry Cultivars and Strains

Blackberry fruits are aggregated fruits composed of several small fruits with longitudinal diameters close to or slightly longer than the transverse diameter. The transverse and longitudinal diameters of the fruits of the 23 cultivars and strains were in the range of 15.83–24.83 cm and 19.72–31.62 cm, respectively (Table 2). The strains and cultivars with the largest transverse diameters were ‘10-5n-2’ and ‘Shuofeng’, and the two cultivars with the largest longitudinal diameters were ‘Kiowa’ and ‘Shuofeng’, indicating that these three blackberry cultivars and strains are large-fruited types. ‘10-5n-2’ had the smallest fruit shape index of 0.99, and ‘Traveler’ had the largest fruit shape index of 1.46. ‘Kiowa’ had the highest fruit weight of 10.43 g, and ‘Young’ had the lowest fruit weight of only 4.05 g. In addition, the fruit weights of ‘Shuofeng’, ‘Black butte’, ‘Shuofeng 2’, and ‘Boysen’ all exceeded 7.0 g.

Among the 23 blackberry cultivars and strains, there were 12 cultivars and 1 hybrid strain with a fruit hardness exceeding 2.0 kg/cm^2^ (Table 2), accounting for more than 50%. ‘Chester’, ‘Ningzhi 2’, and ‘10-5n-2’ had the highest fruit hardness. The fruit hardness of ‘Chester’ was up to 2.78 kg/cm^2^, which gives it the best advantage for staying firm in the consumer market [29,30]. Among the other 10 cultivars and strains with a hardness lower than 2.0 kg/cm^2^, ‘Brazos’ had the lowest hardness of only 1.13 kg/cm^2^.

The coefficient of variation is an index used to measure the difference in a certain characteristic among cultivars and strains, and a larger coefficient of variation reflects the diversity of cultivars and strains for a single characteristic [31]. Through a comprehensive analysis of the differences in five indexes of fruit appearance, including transverse diameter, longitudinal diameter, fruit shape index, fruit weight, and hardness, it was found that the fruit shape index had the smallest difference, with a coefficient of variation of only 10.12%, while the fruit weight had the largest difference, with a coefficient of variation of 23.39%. The difference in fruit hardness was also significant, with a coefficient of variation of 21.46%. Compared to the fruit shape index, blackberry cultivars and strains have a wider diversity in fruit weight and hardness.

### 2.4. Analysis of Fruit Nutritional Characteristics of the 23 Blackberry Cultivars and Strains

The soluble solids content and total acid content of the fruits of the 23 blackberry cultivars and strains ranged from 9.20 to 13.15% and 0.53 to 1.86%, respectively (Table 3). The solid-to-acid ratio is a decisive index of sweetness and sourness. Generally, when the solid-to-acid ratio is between 10 and 15, the sweet and sour degree of the fruit is moderate. Fruits with solid-to-acid ratios above 15 are sweet, and fruits with solid-to-acid ratios below 10 are sour [32]. In this study, the solid-to-acid ratios of the 23 blackberry cultivars and strains ranged from 5.63 to 25.05, among which the solid-to-acid ratios of ‘Ningzhi 2’ and ‘Ningzhi 3’ were the highest, both above 20, indicating that they are both extremely sweet blackberry cultivars. However, the solid-to-acid ratios of ‘Young’ and ‘Boysen’ were the lowest at less than 6, indicating that they are both extremely sour blackberry cultivars.

The three hybrid strains and cultivars with the highest total anthocyanin content among the 23 cultivars and strains were ‘10-5n-2’ (225.4 mg/100 g FW), ‘Shuofeng 2’ (187.7 mg/100 g FW), and ‘Shuofeng’ (171.4 mg/100 g FW), while ‘Young’ had the lowest total anthocyanin content with only 53.95 mg/100 g FW. The three hybrid strains and cultivars with the highest total polyphenol content were ‘10-5n-2’ (3.24 mg/g FW), ‘Shuofeng’ (3.11 mg/g FW), and ‘Chester’ (2.90 mg/g FW). However, ‘Ningzhi 2’ had the lowest total polyphenol content of only 1.82 mg/g FW. Overall, among the five nutritional indexes, the solid-to-acid ratio had the highest coefficient of variation (43.96%).

### 2.5. Correlation, Systematic Clustering and Principal Component Analysis among Various Indicators

**Figure 1:**
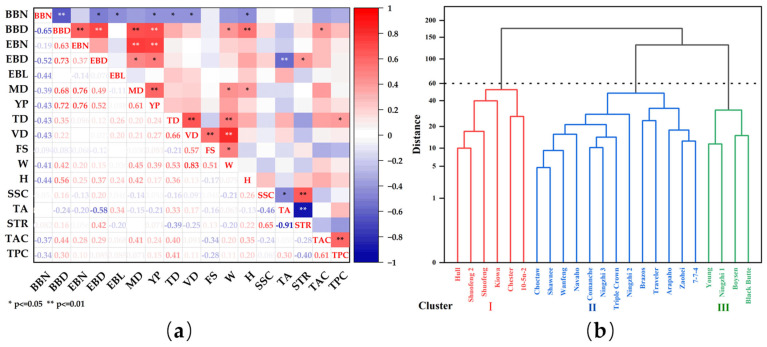
Correlation and cluster analysis of growth, maturity, fruit appearance, and nutritional indexes of 23 blackberry cultivars and strains. (**a**) Correlation heatmap. * Indicates significant correlation *p* < 0.05, ** indicates extremely significant correlation *p* < 0.01. The red square in the figure represents a positive correlation, and the darker the red square is, the stronger the positive correlation. The blue square indicates a negative correlation, and the darker the blue square is, the stronger the negative correlation. BBN, basal branch number; BBD, basal branch diameter; EBN, effective branch number; EBD, effective branch diameter; EBL, effective branch length; MD, maturity duration; YP, yield per plant; TD, transverse diameter; VD, vertical diameter; FS, fruit shape index; W, fruit weight; H, hardness; SSC, soluble solid content; TA, total acid content; STR, solid–acid ratio; TAC, total anthocyanin content; TPC, total polyphenol content. (**b**) The hierarchical cluster diagram divides 23 blackberry cultivars and strains into 3 groups at a distance of 60.

Through correlation analysis of the 17 indexes of blackberry, it was found that among the 8 growth and maturity indexes, the maturity duration was positively correlated with the basal branch diameter and effective branch number (Figure 1a). The yield per plant was positively correlated with the basal branch diameter, effective branch number, and maturity duration. It was concluded that the basal branch diameter, effective branch number, and effective branch diameter jointly affect the maturity duration and plant yield. However, the number of basal branches was negatively correlated with the basal branch diameter and other growth and maturity indexes. Among the 10 appearance quality indexes, the transverse diameter of fruit was significantly positively correlated with fruit weight, the longitudinal diameter was significantly positively correlated with fruit weight and the fruit shape index, and the fruit hardness was significantly positively correlated with the basal branch diameter. The solid-to-acid ratio was positively correlated with the soluble solids content and negatively correlated with the total acid content. There was a highly significant positive correlation between the total anthocyanin content and total polyphenol content.

The 23 blackberry cultivars and strains were divided into 3 groups by hierarchical cluster analysis (Figure 1b). Group I included 5 cultivars and 1 strain: ‘Hull’, ‘Chester’, ‘Kiowa’, ‘Shuofeng’, ‘Shufeng2’, and ‘10-5n-2’. Group II included 12 cultivars and 1 strain: ‘Navaho’, ‘Triple Crown’, ‘Arapaho’, ‘Ningzhi 2’, ‘Ningzhi’, ‘Zaohei’, ‘Choctaw’, ‘Brazos’, ‘Comanche’, ‘Shawnee’, ‘Traveler’, ‘Wanfeng’, and ‘7-7-4’. Group III included 4 cultivars: ‘Young’, ‘Boysen’, ‘Black Butte’ and ‘Ningzhi 1’.

**Figure 2:**
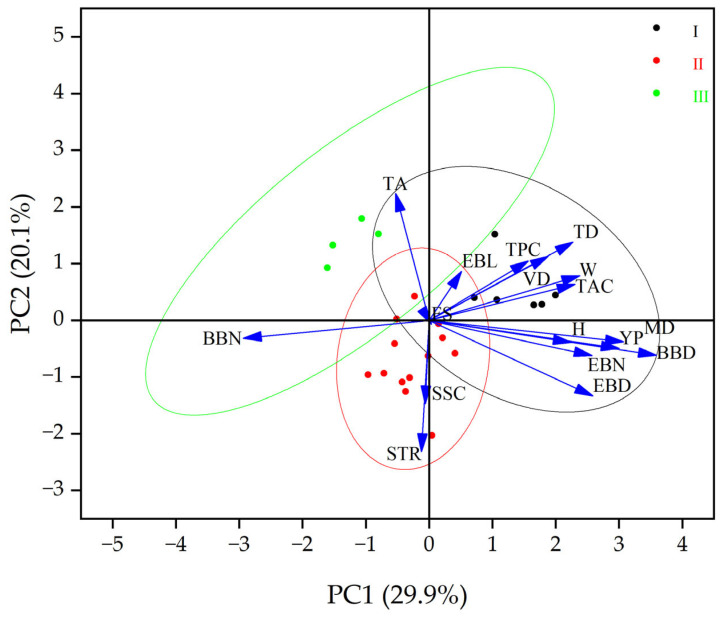
Score and loading plot for principal component analysis (PCA) of the 17 indexes of plant growth and fruit characteristics of the 23 blackberry cultivars and strains. BBN, basal branch number; BBD, basal branch diameter; EBN, effective branch number; EBD, effective branch diameter; EBL, effective branch length; MD, maturity duration; YP, yield per plant; TD, transverse diameter; VD, vertical diameter; FS, fruit shape index; W, fruit weight; H, hardness; SSC, soluble solid content; TA, total acid content; STR, solid–acid ratio; TAC, total anthocyanin content; TPC, total polyphenol content. I, II, and III represent the three phenotype groups by cluster analysis in Figure 1.

Principal component analysis was performed on 17 indicators representing the plant growth and maturity characteristics and fruit appearance and nutritional characteristics of the 23 blackberry cultivars and strains, which were reduced to five principal components, representing a total contribution rate of 79.8% (Figure 2). Among the five principal components, the contribution rate of PC1 was 29.9%, and the indexes of effective branch diameter, effective branch number, maturity duration, and yield per plant had extremely significant positive correlations with PC1, so PC1 can be defined as a growth factor. The contribution rate of PC2 was 20.1%, and the total acid content was positively correlated with PC2, so PC2 can be defined as the acidity factor. The contribution rate of PC3 was 13.3%, and the fruit weight, vertical diameter, and fruit shape index were positively correlated with PC3, so PC3 can be defined as the appearance factor. The contribution rate of PC4 was 10.4%, and the soluble solids content, solid-acid ratio, and hardness were positively correlated with PC4, so PC4 can be defined as the sweetness and hardness factor. The contribution rate of PC5 was 6.1%, and the total polyphenol content was positively correlated with PC5, so PC5 can be defined as an antioxidant activity factor.

Figure 2 shows that most of group I is concentrated in the positive region of PC1 and PC2, indicating that the cultivars and strains in this group have the characteristics of good growth adaptability, but their fruits may have a sour taste. The majority of group II was concentrated in the negative region of PC2, indicating that most of their fruit acidity was relatively low. The cultivars of the third group were concentrated in the negative region of PC1 and the positive region of PC2, which showed that the growth adaptability of the cultivars in group III was poor, the yield was low, and their fruit taste was sour. In addition, when considering the thorn and maturity type of the three groups, it can be found that group I was mainly medium and late maturing cultivars and strains, with a longer maturity duration of 30~45 days, including one thorny cultivar ‘Kiowa’. The second group had the maximum number of 13 cultivars and strains, with a medium maturity duration of 25~35 days, and included 4 thorny cultivars. The third group included all early maturing cultivars, with the shortest maturity duration of 20~25 days, and included 2 thorny cultivars.

### 2.6. Genetic Diversity Analysis by SSR Markers

**Figure 3:**
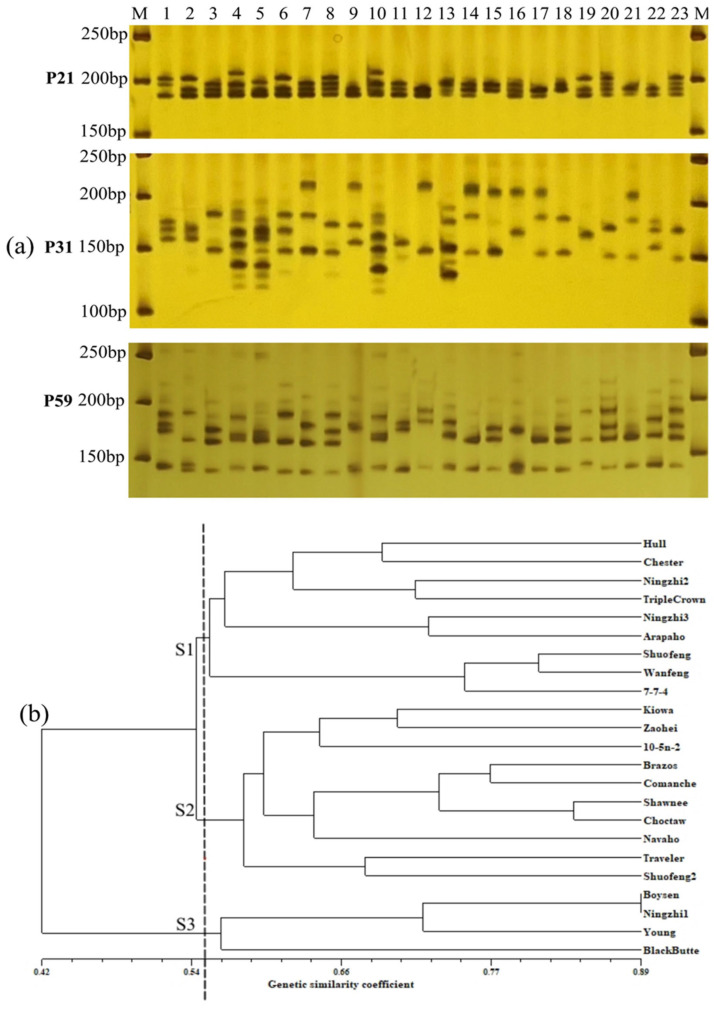
Amplification maps of partial genomic DNA and the UPGMA dendrogram of the 23 blackberry cultivars and strains, (**a**) Three polymorphic primers, P21, P31, and P59, detected five, nine, and seven alleles, respectively. 1, ‘Hull’; 2, ‘Chester’; 3, ‘Kiowa’; 4, ‘Boysen’; 5, ‘Young’; 6, ‘10-5n-2’; 7, ‘Zaohei’; 8, ‘Shuofeng’; 9, ‘Ningzhi 3’; 10,‘Ningzhi 1’; 11, ‘Ningzhi 2’; 12, ‘Arapaho’; 13, ‘Black Butte’; 14, ‘Brazos’; 15, ‘Comanche’; 16, ‘Triple Crown’; 17, ‘Shawnee’; 18, ‘Traveler’; 19,‘Shuofeng 2’; 20, ‘7-7-4’; 21, ‘Choctaw’; 22, ‘Navaho’; 23, ‘Wanfeng’; M, 50 bp DNA ladder. (**b**) Cluster analysis results based on genetic similarity coefficients. The 23 blackberry cultivars and strains were divided into 3 groups, S1, S2, and S3, at a distance of nearly 0.55.

Among the 20 pairs of polymorphic primers, most of the primers amplified 4–8 regions, with relatively rich polymorphism (Table 4). A total of 119 allele variations were detected in the 23 blackberry cultivars and strains, with an average of 6 alleles per SSR locus and a variation range of 2–8. The amplification maps of three pairs of primers, P21, P31, and P59, are shown in Figure 3a, and five, nine, and seven alleles can be detected from clear bands, respectively, among which the number of alleles of P31 is up to nine, and this primer has a strong identification ability among the 23 blackberry cultivars and strains. The polymorphism information content (PIC) was 0.374 (P46)~0.844 (P31), with an average of 0.739, indicating a high genetic diversity. According to the results of the SSR primers, we found that the SSR amplified bands of ‘Boysen’ and ‘Ningzhi 1’ were identical because ‘Ningzhi 1’ is the bud mutation cultivar of ‘Boysen’. The SSR primers P21, P31, and P59 with good polymorphism, clear bands, and high repeatability were selected to construct the fingerprints of the 23 blackberry cultivars and strains (Table S1), and they could completely distinguish 22 blackberry cultivars and strains.

Cluster analysis of the 23 blackberry cultivars and strains using UPGMA (Figure 3b) showed that the 23 cultivars and strains could be divided into 3 genotype groups, S1, S2, and S3, including 9, 10 and 4 blackberry cultivars and strains from top to bottom, respectively. The S1 group contained most of the thornless cultivars and the hybrid F1 generation of parents with no thorns, such as ‘Ningzhi 2’, ‘Ningzhi 3’, and ‘7-7-4’. The S2 group contained most of the thorny cultivars represented by ‘Kiowa’ and the hybrid F1 generation of parents of ‘Kiowa’, such as ‘Zaohei’, ‘10-5n-2’, and ‘Shuofeng 2’. The S3 group included four polyploid blackberry cultivars, ‘Boysen’, ‘Ningzhi 1’, ‘Young’, and ‘Black Butte’. The images of the fruits of the three genotypes can be seen in Figure S1.

### 2.7. Comparison of Fruit Processing Characteristics of 10 Representative Blackberry Cultivars and Strains

On the basis of phenotypic characteristics and genetic diversity analysis, 10 representative blackberry cultivars and strains were selected to investigate fruit processing characteristics. Juice of the different cultivars and strains was prepared by the enzymatic hydrolysis centrifugation method. The differences in juice yield, juice pH, and dry matter content of the 10 blackberry cultivars and strains were compared. Due to the characteristics of the small fruit aggregation of blackberry fruits, their juice mainly exists in the small fruits, so the number of small fruits and the proportion of small fruit weight also affect the processing characteristics; thus, these two indexes were also investigated (Table 5). As shown in Table 4, 5 of the 10 blackberry cultivars and strains had a juice yield of over 70%, which were ‘Chester’, ‘Shuofeng’, ‘Hull’, ‘Kiowa’, and ‘Shuofeng 2’. The pH of the raw juice of the 10 blackberry cultivars and strains was 2.8~3.0, among which the raw juice from ‘Arapaho’, ‘Ningzhi 3’, ‘Shuofeng’, and ‘Shuofeng2’ had pH values of 3.0, while the other cultivars and strains ranged from 2.8 to 2.9.

Among the 10 representative blackberry cultivars and strains, ‘Kiowa’ had the highest number of small fruits (up to 106.8), reaching twice that with the lowest number of small fruits, ‘Chester’ and ’10-5n-2’. ’10-5n-2’ had the highest proportion of small fruits which reached 94.7%, and ‘Wanfeng’ and ‘Chester’ also had high proportions of small fruits, approximately 93~94%, while ‘Zaohei’ had the lowest proportion of small fruits, at only 82.6%. The dry matter contents of the 10 blackberry cultivars and strains ranged from 11.0% to 17.2% (Table 5), with the highest dry matter content being the cultivar with the lowest proportion of small fruits, ‘Zaohei’. Overall, among the five processing characteristics of blackberry fruits, the number of small fruits had the greatest difference, with a coefficient of variation of 28.7%, followed by dry matter content, with a coefficient of variation of 13.3%. The difference in juice yield and juice pH was relatively small, as their coefficient of variation was only slightly over 3%.

## 3. Discussion

‘Hull’ and ‘Chester’ were the first two thornless cultivars of the 23 blackberry cultivars and strains to be planted in the Nanjing area, Jiangsu, China. ‘Kiowa’ had the largest fruit and good growth adaptability, but it was not widely planted because of its thorns. In recent years, the research group has designed multiple hybrid combinations using ‘Kiowa’ as the male or female parent, with the breeding goal focused on having no thorns, high yield, and large fruits. Ultimately, three new cultivars, ‘Shuofeng’ (♀ Kiowa × ♂ Hull), ‘Shuofeng 2’ (♀ Hull × ♂ Kiowa), and ‘Wanfeng’ (♀ Hull × ♂ Kiowa), which have good growth adaptability and high-quality fruits, were obtained. At present, ‘Shuofeng’ is being promoted.

When examining the growth characteristics of blackberry cultivars and strains, it is difficult to ignore the thorns of the plant, as all field management, fruit harvesting, and sample collection work can be inconvenient due to the presence of thorns [33]. Among the first group of cultivars and strains with the best growth adaptability mentioned above, ‘Kiowa’ is a special thorny cultivar. There were also four and two thorny cultivars in the second and third groups, respectively. These 7 thorny blackberry cultivars are all native introduced cultivars, while all 7 independently selected cultivars and 2 hybrid strains among the 23 blackberry cultivars and strains are thornless. This is also a directional result of our research group’s breeding work over the past decade. Fruit ripening characteristics such as maturity duration and plant yield are influenced by growth characteristics. Generally, cultivars and strains with better growth adaptability have a longer maturity duration and higher plant yield, but there are also special cases, such as the hybrid strain ‘10-5n-2’ (♀ Chester × ♂ Kiowa), which has excellent growth adaptability, but fewer inflorescences and a low fruit setting rate, resulting in a lower plant yield. In addition, Vance et al. [34] found that there are certain differences in the flowering and fruit ripening times of different blackberry cultivars in different years, which cannot be predicted using a standard model. This study reached conclusions similar to those of previous studies and found that the same cultivar in the same region may have differences in the timing of fruit ripening due to differences in climate, such as temperature and rainwater. In addition, the plant yield of the cultivars is also affected by various factors such as regional, environmental, and soil conditions, so there may be significant differences between different regions, such as the plant yield of ‘Chester’ in Nanjing being 6.5 kg, while ‘Chester’ planted in Missouri only yielded 3.9 kg per plant [35].

Generally, fruit size is an appearance characteristic that will be of primary concern in the fresh fruit consumer market. However, for soft and juicy blackberry fruits, hardness is also an important index because blackberries are aggregate berries that lack a complete protective cuticle [36]. High hardness can maintain the economic value of blackberry fruits during picking, packaging, transportation, and ultimately entering the fresh fruit consumption market [37]. On the one hand, hardness is affected by the genetic characteristics of the cultivars and strains, such as the F1 generation strain ‘10-5n-2’ with ‘Chester’, as the female parent has the same high hardness as ‘Chester’. On the other hand, the hardness of the fruit is also affected by the external environment, such as light and rain, in the mature period [37]. Adequate light and dry environments are beneficial to the hardness of blackberry fruit and can also improve fruit nutrition, such as sugar and anthocyanin contents [38].

Many consumers, including Chinese consumers, have the impression that blackberry fruits taste sour [39]. However, through the investigation of the fruit quality of the 23 blackberry cultivars and strains, it was found that 14 of the 23 cultivars and strains belonged to moderate or sweet cultivars, accounting for 61%. This is mainly because fully mature fruits were selected during the experimental sampling, while the maturity of blackberry fruits in the consumer market is not sufficient. Furthermore, blackberry cultivars in the consumer market are relatively similar, and the difference in the planting environment of blackberries greatly affects the acidity of blackberry fruits [40,41]. This also reminds us that although blackberry is a vigorous, adaptable, and easily surviving fruit tree in most areas [42], we must not neglect fruit quality while determining yield during cultivation and planting. Adequate light, water, and fertilizer conditions must be provided throughout the entire growth and maturity period of blackberry [43].

The main antioxidant components of blackberries are anthocyanins and polyphenols [21,44,45], while the newly bred strains ‘10-5n-2’ and ‘Shuofeng’ have the highest total polyphenol content, surpassing preliminary research reporting the high polyphenol content of the cultivar ‘Chester’ [21,44]. In addition, the total anthocyanin content of ‘10-5n-2’, ‘Shuofeng 2’, and ‘Shuofeng’ also exceeded that of ‘Chester’, indicating that the blackberry cultivars and strains independently bred by our research group have made important breakthroughs in antioxidant components. Blackberry is a typically processed fruit, and the juice characteristic during processing is the most important characteristic [46]. The pH (2.99) of ‘Chester’ juice grown in China is 9.1% lower than that of ‘Chester’ juice grown in the United States [29], indicating that differences in regional distribution can also cause certain differences in fruit processing characteristics.

Among the 20 pairs of SSR primers selected for the identification of blackberry cultivars and strains in this study, 19 pairs had PIC values exceeding 0.62, indicating good polymorphism and the ability to identify the genetic characteristics of blackberry cultivars and strains accurately and clearly. Three pairs of primers with good polymorphism can effectively distinguish 22 blackberry cultivars and strains, laying a foundation for subsequent resource protection and utilization. From the genetic clustering map of SSR analysis, it can be seen that there is a similar relationship between the hybrid strains and their parents, such as ‘Ningzhi 1’ and ‘Boysen’, ‘Ningzhi 2’ and ‘Triple Crown’, ‘Ningzhi 3’ and ‘Arapaho’, and ‘Zaohei’ and ‘Kiowa’, which is consistent with previous research results [13,15]. However, due to the highly heterozygous genetic background of blackberries, there is a high degree of uncertainty in the performance of hybrid offspring, such as bias toward one parent, parental recombination, and parental heterozygosity [11]. In the hybrid F1 combinations configured with the thorny cultivar ‘Kiowa’ and thornless cultivars ’Hull’, ‘Chester’, and ‘Arapaho’, ‘Shuofeng’ and ‘Wanfeng’ were classified in the thornless S1 group, but ‘Shuofeng2’, ‘10-5n-2’, and ‘Zaohei’ were classified into the thorny S2 group. In addition, by comparing the results of the systematic clustering of phenotypic characters (Figure 1b) and UPGMA clustering based on genetic similarity coefficients (Figure 3b), it was found that the cultivars included in the Class III and S3 groups were completely consistent, indicating that the four cultivars ‘Boysen’, ‘Ningzhi 1’, ‘Young’, and ‘Black Butte’ tend to have lower yields and higher acidity. In this study, 19 out of the 23 blackberry cultivars and strains were tetraploid, accounting for 83%. Among the other four cultivars, ‘Blackbute’ was hexaploid, while ‘Boysen’, ‘Ningzhi 1’, and ‘Young’ were heptaploids [11,47].

## 4. Materials and Methods

### 4.1. Materials

Twenty-three blackberry (*Rubus* spp.) cultivars and strains were planted in the Blackberry Germplasm Repository (119°11′ E, 31°36′ N) of Nanjing Lishui Baima, Institute of Botany, Jiangsu Province and Chinese Academy of Sciences. The annual average temperature of the planting area in 2020 was 17.1 °C, and the annual rainfall was 1294.1 mm. The soil of the resource garden is acidic clay with a pH of approximately 6.0, containing 18.67 g/kg organic matter, 1.25 g/kg total nitrogen, 4.83 mg/kg available phosphorus, and 94.21 mg/kg available potassium. The plants were irrigated under a drip irrigation system. The plant spacing was 1.5 m in rows and 2.5 m between rows, and bird nets were used from June to August during the fruit maturity period.

The 23 blackberry cultivars and strains included 14 introduced cultivars (‘Hull’, ‘Chester’, ‘Choctaw’, ‘Navaho’, ‘Young’, ‘Brazos’, ‘Comanche’, ‘Boysen’, ‘Triple Crown’, ‘Arapaho’, ‘Black Butte’, ‘Kiowa’, ‘Shawnee’, and ‘Traveler’), 4 newly bred cultivars (‘Ningzhi 1’ (bud mutation from ‘Boysen’), ‘Ningzhi 2’ (♀ ‘Triple Crown’ × ♂ ‘Navaho’), ‘Ningzhi 3’ (♀ ‘Arapaho’ × ♂ ‘Hull’), and ‘Shuofeng’ (♀ ‘Kiowa’ × ♂ ‘Hull’)), and 5 hybrid strains (‘Zaohei’ (♀ ‘Kiowa’ × ♂ ‘Arapaho’), ‘Wanfeng’(♀ ‘Hull’ × ♂ ‘Kiowa’), ‘Shuofeng 2’ (♀ ‘Hull’ × ♂ ‘Kiowa’), ‘10-5n-2’ (♀ ‘Chester’ × ♂ ‘Kiowa’), and ‘7-7-4’ (♀ ‘Triple Crown’ × ♂ ‘Chester’)).

Reagents included Folin phenol purchased from Sigma–Aldrich and Klerzyme 150 pectinase purchased from DSM (China) Co., Ltd. (Shanghai, China). The instruments included a PAL-1 Brix meter (Atago China Branch, Guangzhou, China), KM-1 hardness tester (Fujiwara, Tokyo, Japan), Philips HR2166 food processing machine (Philips (China) investment Co., Ltd., Shanghai, China), and ZD-2 automatic potentiometric titrator (Shanghai Jinmai Instrument Co., Ltd. Shanghai, China).

### 4.2. Growth Performance Survey Method

The 4th and 5th year after planting is the most stable period of growth performance, which can reflect the adaptability of the cultivars and strains. Therefore, 4- and 5-year-old plants were selected for the investigation of the 23 cultivars and strains. Referring to the method of Lyu [48], after plant defoliation in winter, the indexes, including the basal branch number, basal branch diameter, effective branch number, effective branch diameter, and effective branch length, were investigated. Eight to ten plants were measured for each cultivar, and the effective branches were fruiting branches with a length of over 50 cm. The fruit ripening time and maturity duration in days of the plants’ 5th and 6th years were recorded, and the fruit yield per plant was calculated [49]. Based on the records of fruit ripening time, the 21 cultivars and 2 strains were classified into early, middle, and late ripening types according to the criteria below: the cultivars and strains that begin to mature in late May or early June are early maturing types, the cultivars and strains that begin to mature in mid or late June are medium maturing types, and the cultivars and strains that begin to mature in late June, early or mid-July are late maturing types.

### 4.3. Method for Determination of Fruit Appearance Indexes

In the full fruit period, 20 fully mature fruits were randomly picked from 4- or 5-year-old plants, and the single fruit weight, transverse diameter, longitudinal diameter, and fruit hardness were measured. The fruits were weighed with an electronic scale with a sensitivity of 0.01 g. The longitudinal and transverse diameters of the fruit were measured using a digital Vernier caliper with a sensitivity of 0.01 mm [50]. The fruit shape index was the ratio of longitudinal diameter to transverse diameter. The hardness was measured using a hardness tester with a probe diameter of 5 mm, which acts on the top of a blackberry fruit to record the force it bears when broken.

### 4.4. Methods for Determination of Fruit Nutritional Indexes

In the full fruit period, approximately 600 g of the fully ripe fruits were hand-harvested once from 4- or 5-year-old plants, frozen into single frozen fruits at −20 °C, and placed in a sealed bag at −20 °C until use. The soluble solids content of 20 single fruits was determined using a Brix meter. The total acid content was determined by acid–base titration, according to GB 12456-2021 Determination of total acid in fruits, and was calculated using citric acid. The ratio of solids to acid was the ratio of soluble solids content to total acid content. The total anthocyanin content was determined by the pH differential method [51]. The total polyphenol content was determined by Folin–Ciocalteu colorimetry [52].

### 4.5. Genetic Diversity Analysis

#### 4.5.1. DNA Extraction and Detection

The apical young leaves of annual nutrition branches of the 23 blackberry cultivars and strains were collected, wrapped with tin foil, quick-frozen in liquid nitrogen, and stored in a −80 °C refrigerator. The blackberry DNA was extracted using the Plant Genome DNA Extraction Kit (Tiangen Company), and the specific operation steps are shown in the instructions. Then, the quality and concentration of DNA were detected by 1% agarose gel electrophoresis and a NanoDrop-1000 spectrophotometer (NanoDrop Technologies, Wilmington, DE, USA), and the final concentration was adjusted to 50 ng/μL^−1^, which was stored at 20 °C for future use.

#### 4.5.2. SSR Marker Analysis

According to published articles related to *Rubus* SSR [53,54] and the SSR primers developed by the research group [11], 20 pairs of primers with good polymorphism were screened and amplified (Table 4, synthesized by Nanjing Qingke Biotechnology Co., Ltd. Nanjing, China) for the next evaluation of the genetic diversity of the 23 blackberry cultivars and strains. The 10 μL PCR system was as follows: 2 μL of 2 × Taq PCR Master Mix, 1 μL of upstream and downstream primers (10 μmol/L), 1 μL of DNA template, and 2 μL of ddH2O. The PCR amplification system was as follows: predenaturation at 95 °C for 2 min, denaturation at 94 °C for 40 s, annealing at 54 °C for 45 s, and extension at 72 °C for 1 min 30 s for 30 cycles. The final extension was at 72 °C for 7 min. The PCR products were separated by 10% polyacrylamide gel electrophoresis (PAGE) and stained with silver nitrate until the bands were clear. The electrophoresis results were read and used for subsequent genetic diversity analysis and fingerprint construction [55,56].

### 4.6. Methods for Determination of Fruit Processing Characteristics

Five hundred grams of the fully ripe fruits were hand-harvested once from 4- or 5-year-old plants in the full fruit period, frozen into single frozen fruits at −18 °C, and kept at −18 °C until use. The fruit juice yield determination was slightly improved with reference to the method of Wang [57]. Approximately 150 g of single frozen fruit was randomly selected for each cultivar or strain, which was naturally thawed at 4 °C for 8 h. After crushing, 0.06% pectinase with a pulp mass fraction was added, and enzymatic hydrolysis was performed at 35 °C for 2 h. Finally, the whole pulp was centrifuged at 5000× *g* r for 5 min, the supernatant juice was weighed, and the juice yield (%) was calculated. The pH value of fruit juice was determined by titrator. Ten fully mature fruits were taken, and tweezers were used to peel the small fruits off one by one from the fruit axis. The number of small fruits was recorded, the weight of the small fruits and the whole fruit was recorded, and the proportion of small fruits was calculated. Five to seven grams of fruit pulp was placed in a water box and dried at 105 °C for more than 8 h until the sample reached a constant weight. The dry matter content (%) of the fruit was calculated. Each sample was repeated 3 times.

### 4.7. Data Processing and Analysis

Origin 2022 (OriginLab Crop., Northampton, MA, USA) software was used to perform hierarchical cluster analysis and principal component analysis. The data before clustering and principal component analysis were standardized by Z scores. The Ward square Euclidean distance clustering method was used for hierarchical cluster analysis, and the correlation coefficient matrix method was used for principal component analysis. In addition, NTSYS-pc Ver.2.10e (Applied Biostatistics Inc., New York, NY, USA) software was used to perform UPGMA-based clustering analysis on the SSR marker information of the 23 blackberry cultivars and strains, and PIC Calc 0.6 software was used to calculate the polymorphism information content (PIC) values of each primer.

## 5. Conclusions

The plant growth and maturity statistics, fruit appearance, nutrition, processing characteristics, and genetic diversity of 23 blackberry cultivars and strains were investigated in the blackberry germplasm repository in Nanjing. The results showed that the variations in plant yield and solid–acid ratio were the largest among the 7 growth indexes and 10 fruit appearance and nutrition indexes. According to phenotypic characteristics, 21 blackberry cultivars and 2 strains can be divided into 3 phenotypes: excellent adaptability, medium adaptability, and poor adaptability. Principal component analysis reduced 17 indexes to 5 principal components: growth, acidity, fruit appearance, sweetness and hardness. In addition, the fingerprints of the 23 blackberry cultivars and strains were constructed using 3 SSR primers, laying a foundation for subsequent resource protection and utilization.

## Figures and Tables

**Table 1:** Growth statistics of different blackberry cultivars and strains.

Cultivar/Strain	Thorns	Basal Branch Number	Basal Branch Diameter/cm	Effective Branch Number	Effective Branch Diameter/cm	Effective Branch Length/cm	Yield per Plant/kg	Ripening Time	Ripening Type	Maturity Duration/d
Ningzhi 1	none	3.0 ± 0.17	1.40 ± 0.08	16 ± 1.6	0.62 ± 0.05	133 ± 12.74	2.8 ± 0.17	Late May to mid-June	Early ripe	25
Ningzhi 3	none	2.8 ± 0.26	1.70 ± 0.09	20 ± 1.92	0.72 ± 0.04	91 ± 7.14	4.8 ± 0.41	Early June to late June	Early ripe	25
Zaohei	none	2.4 ± 0.15	1.90 ± 0.16	17 ± 1.50	0.74 ± 0.04	88 ± 7.64	3.6 ± 0.26	Late May to mid-June	Early ripe	20
Young	none	2.2 ± 0.21	1.35 ± 0.13	11 ± 0.63	0.62 ± 0.03	132 ± 10.79	2.2 ± 0.15	Late May to early June	Early ripe	20
Arapaho	none	3.6 ± 0.35	1.60 ± 0.10	18 ± 1.76	0.71 ± 0.04	56 ± 4.19	2.4 ± 0.22	Late May to mid-June	Early ripe	30
Traveler	none	2.7 ± 0.15	1.80 ± 0.15	16 ± 1.11	0.82 ± 0.07	76 ± 4.35	3.8 ± 0.35	Late May to mid-June	Early ripe	25
Boysen	yes	3.2 ± 0.29	1.50 ± 0.08	16 ± 1.45	0.63 ± 0.05	128 ± 9.91	3.8 ± 0.33	Late May to mid-June	Early ripe	25
Brazos	yes	2.8 ± 0.19	1.60 ± 0.13	28 ± 1.51	0.70 ± 0.04	98 ± 7.97	5.3 ± 0.37	Late May to late June	Early ripe	30
Black Butte	yes	2.2 ± 0.12	1.20 ± 0.06	10 ± 0.99	0.60 ± 0.05	120 ± 6.06	1.3 ± 0.09	Late May to mid-June	Early ripe	20
Choctaw	yes	2.3 ± 0.23	1.80 ± 0.16	21 ± 2.06	0.77 ± 0.04	116 ± 6.22	4.8 ± 0.34	Early June to late June	Early ripe	30
Shawnee	yes	2.5 ± 0.14	1.85 ± 0.10	25 ± 1.36	0.71 ± 0.06	118 ± 10.03	4.5 ± 0.25	Early June to early July	Early ripe	30
Hull	none	2.2 ± 0.20	1.85 ± 0.18	24 ± 2.18	0.78 ± 0.04	126 ± 10.24	5.6 ± 0.45	Mid-June to early July	Medium ripe	30
Shuofeng	none	1.4 ± 0.09	2.30 ± 0.20	18 ± 1.78	0.81 ± 0.05	135 ± 9.20	6.5 ± 0.62	Mid-June to mid-July	Medium ripe	30
Ningzhi 2	none	2.1 ± 0.14	2.10 ± 0.19	22 ± 1.27	0.78 ± 0.07	128 ± 6.61	3.6 ± 0.34	Late June to late July	Medium ripe	30
Triple Crown	none	1.8 ± 0.13	1.90 ± 0.10	15 ± 1.35	0.88 ± 0.08	132 ± 9.01	4.2 ± 0.26	Mid-June to early July	Medium ripe	30
Navaho	none	2.6 ± 0.16	1.75 ± 0.15	18 ± 1.41	0.76 ± 0.05	105 ± 9.68	3.8 ± 0.22	Mid-June to mid-July	Medium ripe	30
Comanche	yes	2.2 ± 0.15	1.80 ± 0.16	20 ± 1.76	0.8 ± 0.06	112 ± 5.72	4.6 ± 0.38	Mid-June to early July	Medium ripe	25
Chester	none	1.8 ± 0.16	2.41 ± 0.15	30 ± 2.01	0.82 ± 0.06	119 ± 6.09	6.5 ± 0.35	Mid July to early August	Late ripe	40
Wanfeng	none	2.2 ± 0.13	1.60 ± 0.12	20 ± 1.63	0.77 ± 0.07	124 ± 10.02	3.5 ± 0.28	Late June to late July	Late ripe	35
Shuofeng 2	none	2.1 ± 0.15	2.10 ± 0.15	22 ± 2.05	0.76 ± 0.04	119 ± 6.23	6.0 ± 0.48	Late June to late July	Late ripe	30
10-5n-2	none	1.9 ± 0.13	2.10 ± 0.13	18 ± 1.41	0.79 ± 0.05	113 ± 9.26	2.3 ± 0.19	Late June to late July	Late ripe	30
7-7-4	none	2.6 ± 0.19	1.70 ± 0.09	19 ± 1.53	0.76 ± 0.04	73 ± 4.72	3.9 ± 0.33	Early July to late July	Late ripe	30
Kiowa	yes	1.6 ± 0.11	2.40 ± 0.21	28 ± 1.88	0.76 ± 0.08	83 ± 5.57	6.3 ± 0.45	Late June to late July	Late ripe	45
Mean		2.4 ± 0.52	1.80 ± 0.32	20 ± 5.02	0.74 ± 0.07	110 ± 22.1	4.2 ± 1.44	-	-	28.9 ± 5.83
Min		1.4	1.2	10	0.6	56	1.3	-	-	20
Max		3.6	2.41	30	0.88	135	6.5	-	-	45
CV/%		22	17.5	25.6	9.67	20.2	34.4	-	-	20.2

Note: Results are expressed as the mean ± standard deviation (SD) (*n* = 3). The same below.

**Table 2:** Differences in fruit appearance characteristics among the 23 blackberry cultivars and strains.

Cultivar/Strain	Transverse Diameter/mm	Vertical Diameter/mm	Fruit Shape Index	Fruit Weight/g	Hardness/kg/cm^2^
Ningzhi 1	17.95 ± 1.56	20.15 ± 1.44	1.12 ± 0.16	4.54 ± 0.53	1.24 ± 0.50
Ningzhi 3	21.25 ± 1.78	24.61 ± 1.32	1.16 ± 0.12	5.87 ± 0.89	2.13 ± 0.17
Zaohei	20.57 ± 1.44	23.11 ± 2.28	1.12 ± 0.12	4.89 ± 1.16	1.72 ± 0.38
Young	18.82 ± 1.39	23.07 ± 2.40	1.23 ± 0.08	4.05 ± 0.75	2.01 ± 0.75
Arapaho	19.26 ± 1.61	23.11 ± 1.91	1.20 ± 0.11	5.08 ± 0.91	2.11 ± 0.33
Traveler	16.22 ± 1.33	23.72 ± 2.66	1.46 ± 0.13	6.86 ± 1.34	1.41 ± 0.28
Boysen	21.79 ± 2.43	28.65 ± 0.94	1.31 ± 0.09	7.03 ± 0.79	1.75 ± 0.21
Brazos	18.63 ± 1.79	24.20 ± 2.02	1.31 ± 0.13	5.73 ± 0.86	1.13 ± 0.20
Black Butte	20.82 ± 1.80	30.68 ± 1.93	1.47 ± 0.11	7.26 ± 1.07	1.73 ± 0.96
Choctaw	18.06 ± 1.76	24.69 ± 2.86	1.37 ± 0.04	5.80 ± 1.21	2.08 ± 0.44
Shawnee	18.39 ± 2.15	22.10 ± 2.05	1.20 ± 0.11	5.40 ± 0.86	2.01 ± 0.25
Hull	23.77 ± 0.95	28.10 ± 1.76	1.24 ± 0.08	6.44 ± 0.78	2.00 ± 0.55
Shuofeng	22.82 ± 1.10	31.02 ± 1.81	1.36 ± 0.13	7.43 ± 1.07	2.07 ± 0.38
Ningzhi 2	17.49 ± 1.08	23.96 ± 2.16	1.37 ± 0.11	4.76 ± 0.78	2.73 ± 0.80
Triple Crown	21.34 ± 1.54	25.22 ± 1.51	1.18 ± 0.10	6.41 ± 0.68	1.99 ± 0.37
Navaho	15.83 ± 0.81	19.72 ± 1.43	1.25 ± 0.02	5.21 ± 0.55	2.06 ± 0.61
Comanche	18.63 ± 1.00	21.57 ± 1.75	1.16 ± 0.11	4.76 ± 0.51	1.56 ± 0.61
Chester	20.94 ± 0.86	22.82 ± 1.38	1.10 ± 0.06	5.48 ± 0.68	2.78 ± 0.51
Wanfeng	20.37 ± 2.00	25.94 ± 4.07	1.28 ± 0.12	5.48 ± 1.27	2.20 ± 0.61
Shuofeng 2	22.13 ± 1.87	26.92 ± 0.92	1.22 ± 0.05	7.05 ± 1.20	2.39 ± 0.65
10-5n-2	24.83 ± 1.84	24.62 ± 2.89	0.99 ± 0.11	6.69 ± 1.16	2.52 ± 0.22
7-7-4	17.98 ± 1.34	21.27 ± 0.99	1.11 ± 0.09	4.27 ± 1.10	1.56 ± 0.82
Kiowa	21.94 ± 1.56	31.62 ± 1.15	1.44 ± 0.12	10.43 ± 0.95	1.98 ± 0.21
Mean	19.99 ± 2.34	24.82 ± 3.33	1.25 ± 0.13	5.95 ± 1.39	1.96 ± 0.42
Min	15.83	19.72	0.99	4.05	1.13
Max	24.83	31.62	1.47	10.43	2.78
CV/%	11.72	13.42	10.12	23.39	21.46

**Table 3:** Differences in fruit nutritional characteristics among the 23 blackberry cultivars and strains.

Cultivar/Strain	Soluble Solid Content/%	Total Acid Content/%	Solid-Acid Ratio	Total Anthocyanin Content (mg/100 g FW)	Total Polyphenol Content (mg/g FW)
Ningzhi 1	10.8 ± 0.28	1.77 ± 0.01	6.12 ± 0.13	102.6 ± 2.40	2.25 ± 0.10
Ningzhi 3	11.8 ± 0.17	0.54 ± 0.01	21.88 ± 0.44	84.6 ± 7.94	2.26 ± 0.13
Zaohei	12.6 ± 0.07	1.23 ± 0.01	10.20 ± 0.25	103.4 ± 5.62	2.83 ± 0.09
Young	10.5 ± 0.14	1.86 ± 0.01	5.63 ± 0.03	54.0 ± 3.60	2.54 ± 0.18
Arapaho	11.7 ± 0.14	0.68 ± 0.01	17.25 ± 0.35	102.5 ± 7.00	2.33 ± 0.13
Traveler	10.1 ± 0.17	0.63 ± 0.02	16.03 ± 0.11	68.4 ± 6.19	2.09 ± 0.09
Boysen	10.3 ± 0.28	1.78 ± 0.08	5.79 ± 0.22	88.7 ± 2.82	2.07 ± 0.13
Brazos	9.2 ± 0.14	1.07 ± 0.03	8.60 ± 0.12	128.0 ± 11.10	2.65 ± 0.08
Black Butte	10.6 ± 0.21	1.42 ± 0.02	7.42 ± 0.07	119.7 ± 6.70	2.51 ± 0.05
Choctaw	11.2 ± 0.17	0.86 ± 0.02	13.04 ± 0.23	138.2 ± 5.02	2.66 ± 0.13
Shawnee	11.1 ± 0.15	0.70 ± 0.03	15.86 ± 0.06	128.9 ± 3.39	2.49 ± 0.10
Hull	11.9 ± 0.28	1.21 ± 0.04	9.83 ± 0.09	94.2 ± 5.44	2.67 ± 0.16
Shuofeng	12.4 ± 0.28	0.88 ± 0.03	14.06 ± 0.45	171.4 ± 2.05	3.11 ± 0.14
Ningzhi 2	13.2 ± 0.17	0.53 ± 0.02	25.05 ± 0.87	71.3 ± 3.60	1.82 ± 0.12
Triple Crown	11.6 ± 0.35	0.71 ± 0.02	16.38 ± 0.26	99.1 ± 3.99	2.22 ± 0.09
Navaho	12.9 ± 0.17	0.70 ± 0.02	18.49 ± 0.72	145.7 ± 13.67	2.72 ± 0.03
Comanche	11.0 ± 0.28	0.62 ± 0.04	17.89 ± 0.12	98.3 ± 9.58	1.93 ± 0.08
Chester	10.1 ± 0.21	1.48 ± 0.05	6.79 ± 0.08	166.5 ± 13.03	2.90 ± 0.10
Wanfeng	10.5 ± 0.35	0.85 ± 0.02	12.37 ± 0.31	143.9 ± 8.30	2.05 ± 0.14
Shuofeng 2	10.8 ± 0.27	1.08 ± 0.03	10.00 ± 0.26	187.7 ± 3.95	2.33 ± 0.04
10-5n-2	9.5 ± 0.36	1.42 ± 0.04	6.71 ± 0.17	225.4 ± 15.41	3.24 ± 0.12
7-7-4	10.9 ± 0.37	1.02 ± 0.01	10.64 ± 0.18	153.1 ± 7.84	2.29 ± 0.06
Kiowa	10.5 ± 0.28	1.22 ± 0.02	8.64 ± 0.05	114.7 ± 7.63	2.30 ± 0.14
Mean	11.07 ± 1.03	1.05 ± 0.41	12.38 ± 5.64	121.3 ± 41.22	2.44 ± 0.37
Min	9.2	0.53	5.63	54.0	1.82
Max	13.2	1.86	25.05	225.4	3.24
CV/%	9.29	39.31	43.96	33.98	14.96

**Table 4:** Twenty pairs of polymorphic SSR primer sequences from *Rubus* spp. and polymorphism detection results.

Primer Name	Repeat Motif	Forward Primer (5′—3′)	Allele Size/bp	Allele Number	PIC Value
P4	(CT)16(CA)32	TGCATGTGACTTTGCATCTCT/GCACTGAAAAATCATGCATCTG	115~200	6	0.677
P5	(CT)7(AT)6(GT)10	ATTCCCCGCCTCAGAATAAT/AAGGTTTGTGACGGGAACAG	123~240	6	0.707
P6	(AT)8(GT)11	TGTTGTACGTGTTGGGCTTT/GGGTGTTTGCCAGTTTCAGT	130~177	6	0.785
P15	(T)10-(A)11-(GA)15	GAGGGGCAATTAAAGGGTTT/TGTTGTAATTTGGTTTATCCTTGG	130~275	8	0.770
P21	(CT)5-(CT)4	CTCACCCGAAATGTTCAACC/GGCTAGGCCGAATGACTACA	185~210	5	0.711
P31	(AG)8	CAGCAGCTAGCATTTTACTGGA/GCACTCTCCACCCATTTCAT	120~210	9	0.844
P40	(TC)9	AACCCTAAGCCAAGGACCAT/CACCACCCATGACAGTCAGA	150~200	5	0.755
P44	(GA)41	TGGACAGCTTTGTGCAGAGT/GCTTGCTTGTATCTCCATTGC	98~155	7	0.816
P46	(TTTTC)3	CATGCTTGCATGATCACCAC/TGAGCCATAAATTTAGAGGGATT	140~150	2	0.374
P48	(GA)10	GCATCAGCCATTGAATTTCC/CCCACCTCCATTACCAACTC	140~187	6	0.756
P55	(AG)15	TGCATGAAGGCGATATAAAGG/TCCGCAAGGGTTGTATCCTA	200~250	7	0.785
P59	(GA)10	GCATCAGCCATTGAATTTCC/CCCACCTCCATTACCAACTC	140~185	7	0.810
P60	(AG)27	CACAACCAGTCCCGAGAAAT/CATTTCATCCAAATGCAACC	107~150	7	0.809
P61	(A)11(AG)8	GCCCCATCCTGTACAAAGAA/TTGCAACAAAGGTACGTAATGG	185~265	5	0.722
Rh37	(AG)10	TTTGGCCCATGTTTGCTCTC/CACTACGCCAAATCAGCTCC	285~320	5	0.763
Rh72	(AAC)8	TTCCGAATCAAGCTCAAAGT/AAACAATAGGTACACGGCTT	325~360	5	0.759
Rh100	(GAGT)5	CCTCACCATCCCACAATTAA/TTTGCTCACCGAATCTGTAT	205~240	4	0.629
Rh114	(TAAT)5	TCGTTCTACACTGTGTTTGT/CGCTGATATCGACTCTGAAT	245~270	4	0.670
Rh118	(TTGGA)5	AGTTTTCCACATGCGTAGAT/TGTACTGCATATTCGAGGAC	140~180	7	0.814
Rh121	(TTGCTC)5bv	AAAAGTCTGTTGGTAGGCAA/TGACTGATGCAAATCTCACA	300~400	8	0.825

**Table 5:** Differences in fruit processing characteristics among 10 excellent blackberry cultivars and strains.

Cultivar/Strain	Yield of Juice/%	pH	Small Fruit Number	Small Fruit Ratio/%	Dry Matter Content/%
Hull	71.0 ± 2.93	2.8 ± 0.05	61.7 ± 5.13	91.5 ± 6.34	14.3 ± 0.41
Chester	72.0 ± 7.06	2.9 ± 0.19	52.3 ± 5.86	93.1 ± 8.68	12.3 ± 1.18
Kiowa	70.6 ± 3.61	2.8 ± 0.05	106.8 ± 11.03	92.4 ± 7.95	14.6 ± 0.04
Shuofeng	71.0 ± 1.47	3.0 ± 0.02	68.0 ± 11.53	92.1 ± 13.02	15.8 ± 0.95
Shuofeng2	70.0 ± 3.82	3.0 ± 0.08	37.0 ± 3.23	92.4 ± 6.72	16.1 ± 0.79
10-5n-2	68.7 ± 6.98	2.9 ± 0.14	51.0 ± 6.93	94.7 ± 10.72	11.0 ± 1.06
Wanfeng	69.0 ± 4.64	2.9 ± 0.08	75.0 ± 2.00	94.5 ± 2.10	14.9 ± 0.21
Ningzhi 3	66.3 ± 1.31	3.0 ± 0.07	58.3 ± 8.06	87.4 ± 10.07	13.1 ± 1.24
Zaohei	64.9 ± 3.45	2.9 ± 0.02	68.8 ± 8.02	82.6 ± 8.03	17.2 ± 1.04
Arapaho	67.9 ± 2.41	3.0 ± 0.13	76.0 ± 5.68	85.4 ± 5.32	16.4 ± 1.53
Mean	69.1 ± 2.25	2.9 ± 0.09	65.5 ± 18.82	90.6 ± 4.06	14.6 ± 1.95
Min	64.9	2.8	37.0	82.6	11.0
Max	72.0	3.0	106.8	94.7	17.2
CV/%	3.26	3.11	28.7	4.48	13.3

## Data Availability

The data and materials supporting the conclusions of this study are included within the article.

## Supplementary Materials

The following supporting information can be downloaded at: https://www.mdpi.com/article/10.3390/plants12162982/s1, Figure S1: Images of the fruits of the three genotypes; Table S1: SSR fingerprint codes of the 23 blackberry cultivars or strains.
