# Supplementary material for: Screening and Evaluation of Excellent Blackberry Cultivars and Strains Based on Nutritional Quality, Antioxidant Properties, and Genetic Diversity
Source: Plants (Basel). 2023 Aug 18;12(16):2982. doi: 10.3390/plants12162982 (PMC10459901; doi:10.3390/plants12162982)
Supplement: Supplementary file 1 [file plants-12-02982-s001.zip › plants-2495979-supplementary.pdf]

Supplementary material

Screening and Evaluation of Excellent Blackberry Cultivars and Strains Based on Nutritional Quality, Antioxidant Properties, and Genetic Diversity

Huifang Zhao [1,2,3], Yaqiong Wu [2], Wenlong Wu [2], Weilin Li [1,*] and Yongcan Jin [1,3*]

1 Co-Innovation Center for Sustainable Forestry in Southern China, College of Forestry, Nanjing Forestry University, 159 Longpan Road, Nanjing 210037, China; zhaohuifang@jib.ac.cn
2 Institute of Botany, Jiangsu Province and Chinese Academy of Sciences (Nanjing Botanical Garden Mem. Sun Yat-Sen), Jiangsu Key Laboratory for the Research and Utilization of Plant Resources, Nanjing 210014, China; yqwu@cnbg.net (Y.W.); wuwenlong@jib.ac.cn (W.W.)
3 Jiangsu Co-Innovation Center of Efficient Processing and Utilization of Forest Resources, College of Light Industry and Food Engineering, Nanjing Forestry University, Nanjing 210037, China
* Correspondence: wlli@njfu.edu.cn (W.L.); jinyongcan@njfu.edu.cn (Y.J.); Tel.: +86-25-8542-8531 (W.L.); +86-25-8542-7636 (Y.J.)

Table S1. SSR fingerprint codes of the 23 blackberry cultivars or strains

Cultivars/strains	SSR fingerprint code
Hull	A185-195-205 B155-160-175 C145-175-180-185
Chester	A185-190-195-205 B155-160 C140-145-165-185
Kiowa	A185-190-195 B150-180 C140-165-175
Boysen	A185-190-195-210 B120-130-140-150-160-180 C145-165-180-185
Young	A185-190 B120-130-140-155-160-175 C145-165-170-175
10-5n-2	A185-190-195-205 B150-160-180 C140-165-185
Zaohei	A185-190-195 B150-180-210 C140-165-175
Shuofeng	A185-190-195-205 B150-175 C140-165-175-185
Ningzhi 3	A185-190-195 B155-175-210 C145-175
Ningzhi 1	A185-190-195-210 B120-130-140-150-160-180 C145-165-180-185
Ningzhi 2	A185-190-195 B155 C145-175
Arapaho	A185-190 B150-210 C145-175-180
Black Butte	A195-205 B140-150-175-180 C145-170-175
Brazos	A185-190-195 B150-175-180-210 C145-165
Comanche	A190-195 B150-210 C145-165
Triple Crown	A185-190-195-205 B160-210 C140-145
Shawnee	A185-190 B150-180-210 C145-165
Traveler	A190-195-205 B150-180 C145-165
Shuofeng 2	A185-190-200 B160 C165-185
7-7-4	A185-190-195-200-205 B150-175 C140-165-185

Choctaw	A185-190 B150-180-210 C140-165
Navaho	A185-190 B155-175-180 C140-145-170-180
Wanfeng	A185-190-195-205 B150-175 C140-165-185

Note: A, B, C represent the marker P21, P31 and P59 respectively.

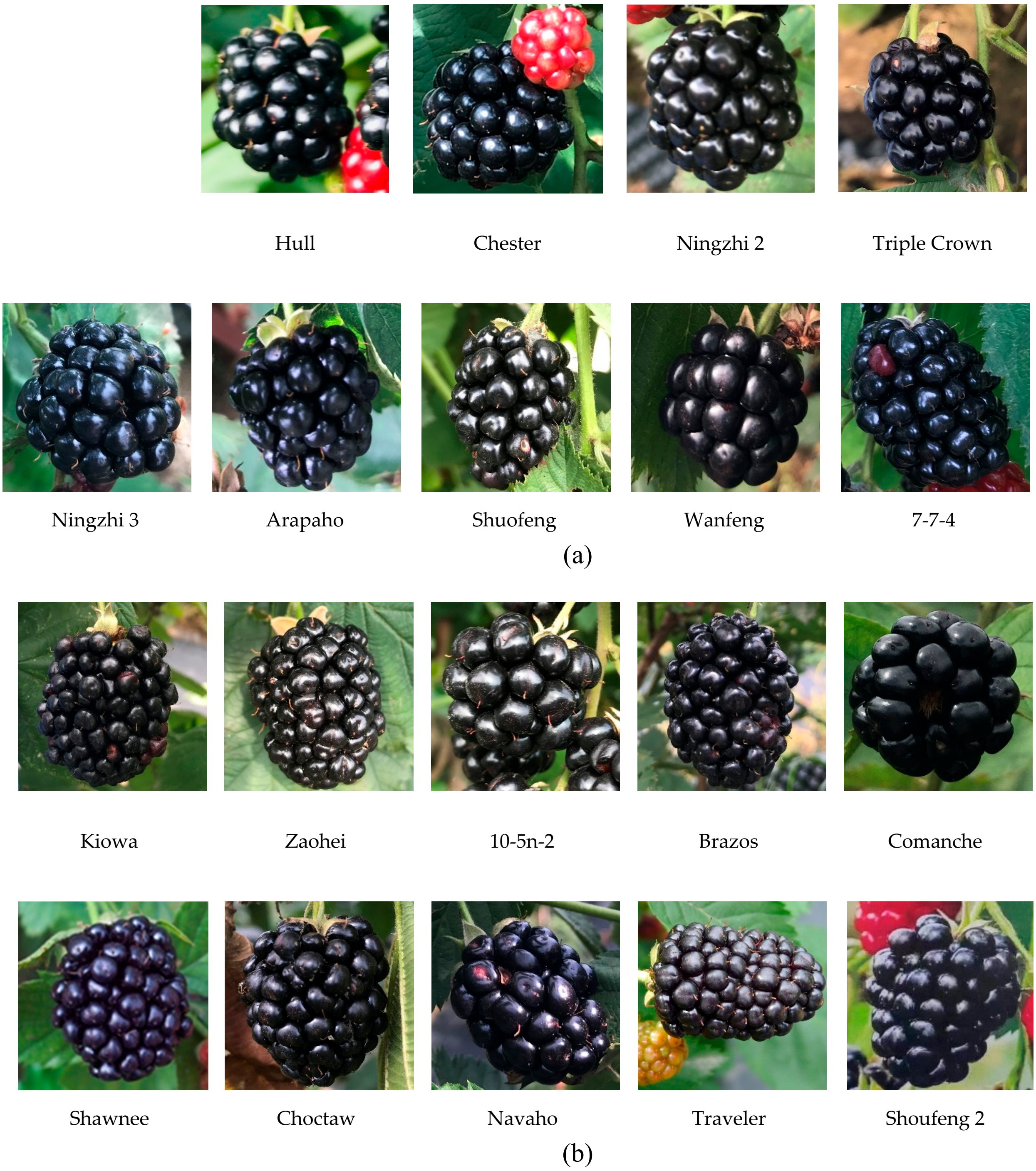

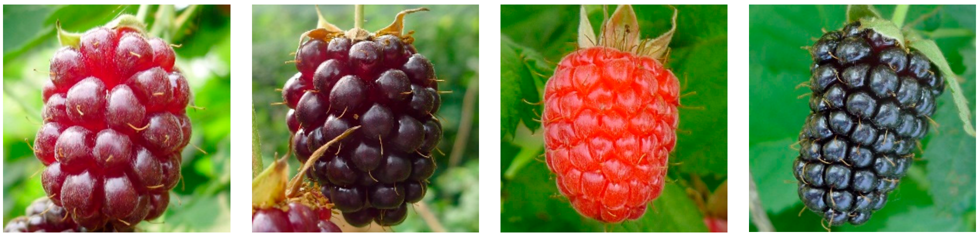

Boysen Ningzhi 1 Young Black Butte

(c)

Figure. S1. Images of the fruits of the three genotypes, (a) refers to S1 group. (b) refers to S2 group. (c) refers to S3 group.
